# Validation of the CancerMath prognostic tool for breast cancer in Southeast Asia

**DOI:** 10.1186/s12885-016-2841-9

**Published:** 2016-10-21

**Authors:** Hui Miao, Mikael Hartman, Helena M. Verkooijen, Nur Aishah Taib, Hoong-Seam Wong, Shridevi Subramaniam, Cheng-Har Yip, Ern-Yu Tan, Patrick Chan, Soo-Chin Lee, Nirmala Bhoo-Pathy

**Affiliations:** 1 Saw Swee Hock School of Public Health, National University of Singapore and National University Health System, Tahir Foundation Building, 12 Science Drive 2, Singapore, 117549 Singapore; 2 Department of Surgery, National University Hospital, 1E Kent Ridge Road, Singapore, 119228 Singapore; 3 Department of Medical Epidemiology and Biostatistics, Karolinska Institutet, PO Box 281, SE-171 77 Stockholm, Sweden; 4 Imaging Division, University Medical Center Utrecht, PO Box 85500, 3508 GA Utrecht, The Netherlands; 5 Department of Surgery, Faculty of Medicine, University of Malaya, 50603 Kuala Lumpur, Malaysia; 6 Clinical Epidemiology Unit, National Clinical Research Centre, Jalan Pahang, 50586 Kuala Lumpur, Malaysia; 7 Department of Surgery, Tan Tock Seng Hospital, 11 Jalan Tan Tock Seng, Singapore, 308433 Singapore; 8 Department of Hematology Oncology, National University Cancer Institute, National University Health System, 1E Kent Ridge Road, Singapore, 119228 Singapore; 9 Department of Social and Preventive Medicine, Faculty of Medicine, University of Malaya, 50603 Kuala Lumpur, Malaysia; 10 Julius Center for Health Sciences and Primary Care, University Medical Center, PO Box 85500, 3508 AB Utrecht, The Netherlands

**Keywords:** Breast cancer, CancerMath, Prognostic model, Asia

## Abstract

**Background:**

CancerMath is a set of web-based prognostic tools which predict nodal status and survival up to 15 years after diagnosis of breast cancer. This study validated its performance in a Southeast Asian setting.

**Methods:**

Using Singapore Malaysia Hospital-Based Breast Cancer Registry, clinical information was retrieved from 7064 stage I to III breast cancer patients who were diagnosed between 1990 and 2011 and underwent surgery. Predicted and observed probabilities of positive nodes and survival were compared for each subgroup. Calibration was assessed by plotting observed value against predicted value for each decile of the predicted value. Discrimination was evaluated by area under a receiver operating characteristic curve (AUC) with 95 % confidence interval (CI).

**Results:**

The median predicted probability of positive lymph nodes is 40.6 % which was lower than the observed 43.6 % (95 % CI, 42.5 %–44.8 %). The calibration plot showed underestimation for most of the groups. The AUC was 0.71 (95 % CI, 0.70–0.72). Cancermath predicted and observed overall survival probabilities were 87.3 % vs 83.4 % at 5 years after diagnosis and 75.3 % vs 70.4 % at 10 years after diagnosis. The difference was smaller for patients from Singapore, patients diagnosed more recently and patients with favorable tumor characteristics. Calibration plot also illustrated overprediction of survival for patients with poor prognosis. The AUC for 5-year and 10-year overall survival was 0.77 (95 % CI: 0.75–0.79) and 0.74 (95 % CI: 0.71–0.76).

**Conclusions:**

The discrimination and calibration of CancerMath were modest. The results suggest that clinical application of CancerMath should be limited to patients with better prognostic profile.

## Background

Adjuvant chemotherapy and hormone therapy improve long-term survival and reduce the risk of recurrence in early breast cancer patients [1–3]. However, the benefit varies greatly from patient to patient due to biologic heterogeneity of the disease and differences in response to treatment [4, 5]. Risk of adverse effects and high cost of adjuvant therapy also make it challenging for oncologists to choose the most appropriate treatment. Therefore, several clinical tools have been developed to predict prognosis and survival benefit from treatment, using clinicopathological features, genetic profiles, and novel biomarkers [6].

The Nottingham Prognostic Index was the first prognostic model introduced for breast cancer patients in 1982. It includes only tumor grade, size, and nodal status for prediction of disease-free survival [7, 8]. The widely used Adjuvant! Online (www.adjuvantonline.com) calculates 10-year overall survival and disease-free survival of patients with non-metastatic breast cancer, based on patient’s age, tumor size, grade, estrogen-receptor (ER) status, nodal status, and co-morbidities. It also quantitatively predicts the absolute gain from adjuvant therapy [9]. Although it is recommended by the National Institute for Health and Clinical Excellence and widely used by oncologists [10–13], several validation studies have suggested that Adjuvant! Online is suboptimal in women younger than 40 years and older than 75 years [14, 15]. The model was recently validated in Malaysia, Korea, and Taiwan, where it was shown to substantially overestimate actual survival [16–18]. CancerMath (http://www.lifemath.net/cancer/) is the latest web-based prognostic tool, which takes human epidermal growth factor receptor 2 (HER2) status into account [19]. It was established based on the binary biological model of cancer metastasis and the parameters were derived from the Surveillance, Epidemiology and End-Result (SEER) registry in the United States [20]. CancerMath provides information on overall survival, conditional survival (the likelihood of surviving given being alive after a certain number of years) and benefit of systemic treatment for each of the first 15 years after diagnosis. This model also estimates probability of positive lymph nodes and nipple involvement. Validation study has shown comparable results between CancerMath and Adjuvant! Online [19]. However this new tool has not been validated outside the United States. Given the differences in underlying distribution of prognostic factors and life expectancy between Asia and the United States [21–23], direct application without any correction may not generate reliable prediction. The aim of the study is to validate this model in the Singapore Malaysia Hospital-Based Breast Cancer Registry, demonstrating its predictive performance for different subgroups and determining its calibration and discrimination.

## Methods

Women diagnosed with pathological stage I to III breast cancer according to American Joint Committee on Cancer Staging Manual sixth edition, who underwent surgery, were identified from the Singapore Malaysia Hospital-Based Breast Cancer Registry, which combined databases from three public tertiary hospitals. The breast cancer registry at National University Hospital (NUH) in Singapore collects information on breast cancer patients diagnosed since 1990. The Tan Tock Seng Hospital (TTSH) registry registers patients diagnosed from 2001 onwards. The University Malaya Medical Centre (UMMC), located in Kuala Lumpur, Malaysia, has prospectively collected data on breast cancer patients diagnosed since 1993 [24]. No consent was needed and ethics approval was obtained from Domain Specific Review Board under National Healthcare Group in Singapore and Medical Ethics Committee under UMMC. The consolidated registry included information on ethnicity, age and date of diagnosis, histologically determined tumor size, number of positive lymph nodes, ER and progesterone receptor (PR) status (positive defined as 1 % or more positively stained tumor cells at NUH or 10 % or more positively stained tumor cells at TTSH and UMMC, negative, or unknown), HER2 status based on fluorescence in situ hybridization (FISH) or immunohistochemistry (IHC) if FISH was not performed (positive defined as FISH positive or IHC score of 3+, negative defined as FISH negative or IHC scored of 0 or 1+, equivocal defined as IHC score of 2+, or unknown), histological type (ductal, lobular, mucinous, others, or unknown), grade (1, 2, 3, or unknown), type of surgery (no surgery, mastectomy, breast conserving surgery, or unknown), chemotherapy (yes, no or unknown), hormone therapy (yes, no, or unknown), and radiotherapy (yes, no, or unknown). Detailed chemotherapeutic treatment regimens were only available for UMMC patients. For chemotherapy, cyclophosphamide, methotrexate and fluorouracil (CMF) was categorized as first generation regimen and fluorouracil, epirubicin and cyclophosphamide (FEC), and doxorubicin and cyclophosphamide (AC) followed by paclitaxel were second generation. Docetaxel, doxorubicin and cyclophosphamide (TAC), and FEC followed by docetaxel were categorized as third generation. Hormone therapy was categorized into five groups: tamoxifen, aromatase inhibitors (AI), tamoxifen followed by AI, ovarian ablation, and ovarian ablation plus tamoxifen. Vital status was obtained from the hospitals' medical records and ascertained by linkage to death registries in both countries. Patients diagnosed until 31^st^ December 2011 were followed up from date of diagnosis until date of death or date of last fellow-up, whichever came first. Date of last follow-up was 1^st^ March 2013 for UMMC, 31^st^ July 2013 for NUH, and 1^st^ October 2012 for TTSH. Male patients, patients with unknown age at diagnosis and tumor size were excluded from this analysis as these two were essential predictors for all four CancerMath calculators.

Javascript codes of all four CancerMath calculators which contained predetermined parameters and mathematical equations were exported on 9^th^ Nov 2013 from its website by selecting “view- > source” in the browser menu. The script was then transcribed into R script to allow calculation for a group of patients. For nodal status calculator, patient’s age, tumor size, ER and PR status, histological type, and grade were used by the program to calculate probability of positive nodes for each patient. Overall mortality risk at each year up to 15 year after diagnoses was predicted by outcome calculator, based on age, tumor size, number of positive nodes, grade, histological type, ER, PR, and HER2 status. Effect of hormone and chemotherapeutic regimen on overall mortality was further adjusted by the therapy calculator and number of years since diagnosis were considered in the conditional survival calculator. Results from R script and website were crosschecked with a random subset of 20 patients to verify the accuracy of R script. Histological type recorded as others was re-categorized as unknown. If HER2 status was equivocal based on IHC and FISH was not performed, HER2 status was treated as unknown. Evidence of recurrence was set as unknown for conditional survival calculation.

In total, 7064 female breast cancer patients were included. Only cases with known nodal status (*N* = 6807) were included for validation of nodal status calculator and their individual probability of positive lymph nodes was calculated. For outcome calculator, two separate subsets of patients with minimum 5-year follow up (UMMC and NUH patients diagnosed in 2007 and earlier and TTSH patient diagnosed in 2006 and earlier, *N* = 4517) and patients with 10-year follow-up UMMC and NUH cases diagnosed in 2002 and earlier, *N* = 1649) were selected for comparison of observed and predicted survival. As NUH and TTSH did not collect details of hormone therapy and chemotherapy regimen data before 2006, therapy calculator was only validated for UMMC patients with minimum 5-year follow up (*N* = 1538).

### Statistical analysis

#### Nodal status calculator

Observed and predicted probability of positive lymph nodes were compared. Calibration was assessed by dividing the data into deciles based on the predicted probability of positive nodes and then plotting the observed probability of positive nodes against means of predicted probability for each decile. A 45 degree diagonal line was plotted to illustrate perfect agreement. Discrimination of nodal status calculator was evaluated by area under the curve (AUC) in receiver operating characteristic analysis. A value of 0.5 indicates no discrimination and a value of 1.0 means perfect discrimination.

#### Outcome and therapy calculator

Ratio of observed and predicted numbers of death within 5 years and 10 years of diagnosis were calculated as mortality ratio (MR) with 95 % confidence interval (CI) constructed by exact procedure [25]. MR was also calculated for different subgroups by country, period of diagnosis, age, race, and other clinical characteristics. Observed 5-year and 10-year survival rates were compared with the median predicted survival from CancerMath. A difference of less than 3 % would be considered reliable enough for clinical use as 10-year survival benefit of 3–5 % is an indication for adjuvant chemotherapy [26]. The relationship of average 5-year and 10-year predicted survival and observed 5-year and 10-year survival was illustrated by the calibration plot. Discrimination of outcome and therapy calculator was evaluated by AUC using dataset with minimum 5-year and 10-year follow-up accordingly. Outcome calculator was further evaluated using concordance index (c-index) proposed by Harrell et al. for the entire dataset regardless of follow-up time [27]. C-index is the probability of correctly distinguishing patient who survives longer within a random pair of patients [27]. Like for the AUC, a c-index of 0.5 indicates no discrimination and a c-index of 1.0 means perfect discrimination.

#### Conditional survival calculator

For patients who survived two years after diagnosis, predicted 5-year survival was compared with observed 5-year survival. Similarly predicted 10-year survival was compared with observed 10-year survival for patients who survived 5 years and 7 years respectively. Discrimination was evaluated by AUC.

## Results

In total, 7064 female breast cancer patients were included. Tables 1, 2, 3 and 4 present clinical characteristics of 6807 patients with nodal status, 4517 patients with minimum 5-year follow-up, 1649 patients with 10-year follow-up, and 1538 patients with detailed treatment data and minimum of 5-years follow-up, respectively.

**Table 1:** Observed number of patients with positive lymph nodes and predicted probability of positive nodes

	Number of patients	Number of patients with positive lymph nodes (percentage)	Predicted probability of positive nodes (median)
Overall	6807	2970 (43.6 %)	40.6 %
Ethnicity			
Chinese	5029	2062 (41.0 %)	39.2 %
Malay	963	511 (53.1 %)	46.0 %
Indian	651	312 (47.9 %)	44.7 %
Other	164	85 (51.8 %)	39.5 %
Country			
Malaysia	3274	1460 (44.6 %)	43.0 %
Singapore	3533	1510 (42.7 %)	38.5 %
Period of diagnosis			
1990–1994	124	58 (46.8 %)	52.0 %
1995–1999	547	258 (47.2 %)	41.9 %
2000–2003	1744	755 (43.3 %)	41.4 %
2004–2007	2129	964 (45.3 %)	41.2 %
2008–2011	2263	935 (41.3 %)	38.9 %
Age at diagnosis			
0–39	670	310 (46.3 %)	47.1 %
40–49	2039	910 (44.6 %)	42.9 %
50–59	2145	934 (43.5 %)	41.4 %
60–69	1301	546 (42.0 %)	36.7 %
70+	652	270 (41.4 %)	34.3 %
Tumor size (mm)			
0–20	2926	822 (28.1 %)	26.4 %
21–50	3247	1678 (51.7 %)	49.3 %
51+	634	470 (74.1 %)	79.2 %
ER status			
Negative	2316	1037 (44.8 %)	43.5 %
Positive	4254	1854 (43.6 %)	38.5 %
Unknown	237	79 (33.3 %)	44.5 %
PR status			
Negative	2656	1195 (45.0 %)	42.1 %
Positive	3507	1511 (43.1 %)	38.5 %
Unknown	644	264 (41.0 %)	44.2 %
Her2 status			
Negative	2872	1197 (41.7 %)	39.2 %
Equivocal	429	182 (42.4 %)	39.2 %
Positive	1315	662 (50.3 %)	45.0 %
Unknown	2191	929 (42.4 %)	39.6 %
Histology			
Ductal	5945	2681 (45.1 %)	41.5 %
Lobular	287	150 (52.3 %)	37.9 %
Mucinous	219	34 (15.5 %)	10.7 %
Others	352	102 (29.0 %)	35.8 %
Unknown	4	3 (75.0 %)	25.1 %
Grade			
1	849	204 (24.0 %)	21.8 %
2	2836	1278 (45.1 %)	40.6 %
3	2463	1275 (51.8 %)	46.4 %
Unknown	659	213 (32.3 %)	35.9 %

**Table 2:** Observed and predicted 5-year overall survival from outcome calculator, stratified by patients’ characteristics

	*N*	Observed deaths in 5 years	Predicted deaths in 5 years	Mortality Ratio (95 % CI)	Observed 5-year survival (%) (std err)	Predicted 5-year survival (median) (%)	Absolute difference (%) (95 % CI)
Overall	4517	752	667	1.13(1.05,1.21)	83.4 (0.006)	87.3	**3.9 (2.7,5.1)**
Ethnicity							
Chinese	3340	488	478	1.02(0.93,1.12)	85.4 (0.006)	88.0	**2.6 (1.4,3.8)**
Malay	654	143	104	**1.38(1.16,1.62)**	78.1 (0.016)	85.8	**7.7 (4.6,10.8)**
Indian	430	109	71	**1.54(1.26,1.85)**	74.7 (0.021)	85.1	**10.4 (6.3,14.5)**
Other	93	12	14	0.86(0.44,1.50)	87.1 (0.035)	87.3	0.2 (−6.7,7.1)
Country							
Malaysia	2143	423	331	**1.28(1.16,1.41)**	80.3 (0.009)	86.1	**5.8(4.0,7.6)**
Singapore	2374	329	336	**0.98(0.88,1.09)**	86.1 (0.007)	88.6	**2.5(1.1,3.9)**
Period of diagnosis							
1990–1994	140	41	22	**1.86(1.34,2.53)**	70.7 (0.038)	85.9	**15.2 (7.8,22.6)**
1995–1999	564	116	75	**1.55(1.28,1.86)**	79.8 (0.017)	87.9	**8.1 (4.8,11.4)**
2000–2003	1800	279	261	1.07(0.95,1.20)	84.5 (0.009)	87.8	**3.3 (1.5,5.1)**
2004–2007	2013	316	309	1.02(0.91,1.14)	84.3 (0.008)	87.2	**2.9 (1.3,4.5)**
Age at diagnosis							
0–39	493	101	64	**1.58(1.29,1.92)**	79.5 (0.018)	88.8	**9.3(5.8,12.8)**
40–49	1430	172	163	1.06(0.90,1.23)	88.0 (0.009)	90.6	**2.6(0.8,4.4)**
50–59	1412	224	194	**1.15(1.01,1.32)**	84.1 (0.010)	88.2	**4.1(2.1,6.1)**
60–69	776	126	130	0.97(0.81,1.15)	83.8 (0.013)	85.1	1.3(−1.2,3.8)
70+	406	129	117	1.10(0.92,1.31)	68.2 (0.023)	73.9	**5.7 (1.2,10.2)**
Tumor size (mm)							
0–20	1889	151	173	0.87(0.74,1.02)	92.0 (0.006)	92.9	0.9(−0.3,2.1)
21–50	2180	438	374	**1.17(1.06,1.29)**	79.9 (0.009)	84.8	**4.9 (3.1,6.7)**
51+	448	163	121	**1.35(1.15,1.57)**	63.6 (0.023)	73.6	**10.0(5.5,14.5)**
Number of positive nodes							
0	2408	196	238	**0.82(0.71,0.95)**	91.9 (0.006)	91.7	−0.2(−1.4,1.0)
1–3	1068	195	165	**1.18(1.02,1.36)**	81.7 (0.012)	85.9	**4.2(1.8,6.6)**
4–9	533	159	122	**1.30(1.11,1.52)**	70.2 (0.020)	78.0	**7.8(3.9,11.7)**
10+	354	170	116	**1.47(1.25,1.70)**	52.0 (0.027)	67.4	**15.4(10.1,20.7)**
Unknown	154	32	27	1.19(0.81,1.67)	79.2 (0.033)	86.6	**7.4(0.9,13.9)**
ER status							
Negative	1595	392	268	**1.46(1.32,1.61)**	75.4 (0.011)	85.2	**9.8 (7.6,12.0)**
Positive	2668	309	367	**0.84(0.75,0.94)**	88.4 (0.006)	88.8	0.4(−0.8,1.6)
Unknown	254	51	33	**1.55(1.15,2.03)**	79.9 (0.025)	88.6	**8.7(3.8,13.6)**
PR status							
Negative	1674	382	289	**1.32(1.19,1.46)**	77.2 (0.010)	84.8	**7.6(5.6,9.6)**
Positive	2174	241	285	**0.85(0.74,0.96)**	88.9 (0.007)	89.5	0.6(−0.8,2.0)
Unknown	669	129	93	**1.39(1.16,1.65)**	80.7 (0.015)	87.4	**6.7 (3.8,9.6)**
Her2 status							
Negative	1483	208	210	0.99(0.86,1.13)	86.0 (0.009)	88.0	**2.0(0.2,3.8)**
Equivocal	118	19	19	1.00(0.60,1.56)	83.9 (0.034)	87.4	3.5(−3.2,10.2)
Positive	790	172	147	**1.17(1.00,1.36)**	78.2 (0.015)	83.0	**4.8(1.9,7.7)**
Unknown	2126	353	292	**1.21(1.09,1.34)**	83.4 (0.008)	88.7	**5.3(3.7,6.9)**
Histology							
Ductal	3951	696	597	**1.17(1.08,1.26)**	82.4 (0.006)	87.0	**4.6 (3.4,5.8)**
Lobular	180	17	26	0.65(0.38,1.05)	90.6 (0.022)	87.5	−3.1(−7.4,1.2)
Mucinous	156	10	14	0.71(0.34,1.31)	93.6 (0.020)	94.7	1.1(−2.8,5.0)
Others	227	29	30	0.97(0.65,1.39)	87.2 (0.022)	89.5	2.3(−2.0,6.6)
Unknown	3	0	0	-	100	86.8	−13.2
Grade							
1	552	20	44	**0.45(0.28,0.70)**	96.4 (0.008)	94.7	**−1.7(−3.3,–0.1)**
2	1882	261	265	0.98(0.87,1.11)	86.1 (0.008)	88.2	**2.1(0.5,3.7)**
3	1591	402	288	**1.40(1.26,1.54)**	74.7 (0.011)	84.3	**9.6(7.4,11.8)**
Unknown	492	69	70	0.99(0.77,1.25)	86.0 (0.016)	87.4	1.4(−1.7,4.5)

Numbers marked in bold indicate statistically significant difference at the 95% confidence level

**Table 3:** Observed and predicted 10-year overall survival from outcome calculator, stratified by patients’ characteristics

	*N*	Observed death in 10 years	Predicted death in 10 years	Mortality Ratio (95 % CI)	Observed 10-year survival (%)(std err)	Predicted 10-year survival (median) (%)	Absolute difference (%) (95 % CI)
Overall	1649	488	454	1.07(0.98,1.17)	70.4 (0.011)	75.3	**4.9 (2.7,7.1)**
Ethnicity							
Chinese	1201	318	318	1.00(0.89,1.12)	73.5 (0.013)	76.8	**3.3 (0.8,5.8)**
Malay	251	100	74	**1.35(1.10,1.64)**	60.2 (0.031)	72.3	**12.1(6.0,18.2)**
Indian	174	64	55	1.16(0.90,1.49)	63.2 (0.037)	69.9	6.7 (−0.6,14.0)
Other	23	6	7	0.86(0.31,1.87)	73.9 (0.092)	77.1	3.2 (−14.8,21.2)
Country							
Malaysia	983	341	284	**1.20(1.08,1.34)**	65.3 (0.015)	73.3	**8.0 (5.1,10.9)**
Singapore	666	147	170	0.86(0.73,1.02)	77.9 (0.016)	77.9	0.0 (−3.1,3.1)
Period of diagnosis							
1990–1994	140	56	42	**1.33(1.01,1.73)**	60.0 (0.041)	72.5	**12.5(4.5,20.5)**
1995–1999	564	187	148	**1.26(1.09,1.46)**	66.8 (0.020)	76.0	**9.2 (5.3,13.1)**
2000–2002	945	245	264	0.93(0.82,1.05)	74.1 (0.014)	75.9	1.8 (−0.9,4.5)
Age at diagnosis							
0–39	232	82	58	**1.41(1.12,1.75)**	64.7 (0.031)	77.3	**12.6 (6.5,18.7)**
40–49	576	137	130	1.05(0.88,1.25)	76.2 (0.018)	80.2	**4.0 (0.5,7.5)**
50–59	493	141	129	1.09(0.92,1.29)	71.4 (0.020)	76.4	**5.0 (1.1,8.9)**
60–69	254	78	86	0.91(0.72,1.13)	69.3 (0.029)	68.4	−0.9 (−6.6,4.8)
70+	94	50	50	1.00(0.74,1.32)	46.8 (0.051)	50.1	3.3 (−6.7,13.3)
Tumor size (mm)							
0–20	653	118	109	1.08(0.90,1.30)	81.9 (0.015)	86.8	**4.9 (2.0,7.8)**
21–50	831	283	262	1.08(0.96,1.21)	65.9 (0.016)	70.6	**4.7 (1.6,7.8)**
51+	165	87	82	1.06(0.85,1.31)	47.3 (0.039)	50.6	3.3 (–4.3,10.9)
Number of positive nodes							
0	867	147	161	0.91(0.77,1.07)	83.0 (0.013)	84.0	1.0 (−1.5,3.5)
1–3	407	143	120	**1.19(1.00,1.40)**	64.9 (0.024)	72.1	**7.2 (2.5,11.9)**
4–9	215	112	93	1.20(0.99,1.45)	47.9 (0.034)	58.2	**10.3 (3.6,17.0)**
10+	104	71	62	1.15(0.89,1.44)	31.7 (0.046)	39.9	8.2 (−0.8,17.2)
Unknown	56	15	17	0.88(0.49,1.46)	73.2 (0.059)	73.5	0.3 (−11.3,11.9)
ER status							
Negative	637	224	197	1.14(0.99,1.30)	64.8 (0.019)	71.5	**6.7 (3.0,10.4)**
Positive	816	205	206	1.00(0.86,1.14)	74.9 (0.015)	78.2	**3.3 (0.4,6.2)**
Unknown	196	59	51	1.16(0.88,1.49)	69.9 (0.033)	76.8	**6.9 (0.4,13.4)**
PR status							
Negative	485	160	153	1.05(0.89,1.22)	67.0 (0.021)	70.7	3.7(−0.4,7.8)
Positive	564	128	136	0.94(0.79,1.12)	77.3 (0.018)	79.9	2.6 (−0.9,6.1)
Unknown	600	200	165	**1.21(1.05,1.39)**	66.7 (0.019)	74.1	**7.4 (3.7,11.1)**
Her2 status							
Negative	269	72	66	1.09(0.85,1.37)	73.2 (0.027)	78.3	5.1(−0.2,10.4)
Equivocal	13	6	4	1.50(0.55,3.26)	53.8 (0.138)	65.5	11.7 (−15.3,38.7)
Positive	335	113	110	1.03(0.85,1.24)	66.3 (0.026)	69.1	2.8 (−2.3,7.9)
Unknown	1032	297	273	1.09(0.97,1.22)	71.2 (0.014)	76.8	**5.6 (2.9,8.3)**
Histology							
Ductal	1418	445	401	**1.11(1.01,1.22)**	68.6 (0.012)	74.4	**5.8 (3.4,8.2)**
Lobular	78	18	21	0.86(0.51,1.35)	76.9 (0.048)	75.7	−1.2 (−10.6,8.2)
Mucinous	59	9	9	1.00(0.46,1.90)	84.7 (0.047)	91.2	6.5 (−2.7,15.7)
Others	91	16	22	0.73(0.42,1.18)	82.4 (0.040)	77.7	−4.7 (−12.5,3.1)
Unknown	3	0	1	-	100	74.4	−25.6
Grade							
1	200	22	31	0.71(0.44,1.07)	89.0 (0.022)	89.3	0.3 (−4.0,4.6)
2	668	188	176	1.07(0.92,1.23)	71.9 (0.017)	77.1	**5.2 (1.9, 8.5)**
3	510	196	172	1.14(0.99,1.31)	61.6 (0.022)	70.0	**8.4 (4.1,12.7)**
Unknown	271	82	76	1.08(0.86,1.34)	69.7 (0.028)	73.3	3.6 (−1.9,9.1)

Numbers marked in bold indicate statistically significant difference at the 95% confidence level

**Table 4:** Observed and predicted 5-year overall survival from therapy calculator, stratified by patients’ characteristics

	*N*	Observed death in 5 years	Predicted death in 5 years	Mortality Ratio (95 % CI)	Observed 5-year survival (%)(std err)	Predicted 5-year survival (median) (%)	Absolute difference (%) (95 % CI)
Overall	1538	286	173	**1.65(1.47,1.86)**	81.4 (0.010)	89.8	**8.4(6.4,10.4)**
Ethnicity							
Chinese	1052	167	113	**1.48(1.26,1.72)**	84.1 (0.011)	90.4	**6.3(4.1,8.5)**
Malay	264	62	30	**2.07(1.58,2.65)**	76.5 (0.026)	89.4	**12.9(7.8,18.0)**
Indian	212	54	29	**1.86(1.40,2.43)**	74.5 (0.030)	87.2	**12.7(6.8,18.6)**
Other	10	3	1	3.00(0.62,8.77)	70.0 (0.145)	88.2	18.2(−10.2,46.6)
Period of diagnosis							
1990–1994	95	39	14	**2.79(1.98,3.81)**	58.9 (0.05)	86.8	**27.9 (18.1,37.7)**
1995–1999	374	93	40	**2.33(1.88,2.85)**	75.1 (0.022)	90.9	**15.8 (11.5,20.1)**
2000–2003	568	91	63	**1.44(1.16,1.77)**	84.0 (0.015)	89.7	**5.7 (2.8,8.6)**
2004–2007	501	63	56	1.13(0.86,1.44)	87.4 (0.015)	90.2	2.8 (−0.1,5.7)
Age at diagnosis							
0–39	205	55	17	**3.24(2.44,4.21)**	73.2 (0.031)	92.6	**19.4(13.3,25.5)**
40–49	515	74	41	**1.80(1.42,2.27)**	85.6 (0.015)	92.9	**7.3 (4.4,10.2)**
50–59	449	86	50	**1.72(1.38,2.12)**	80.8 (0.019)	89.4	**8.6 (4.9,12.3)**
60–69	271	43	40	1.08(0.78,1.45)	84.1 (0.022)	86.1	2.0 (−2.3,6.3)
70+	98	28	24	1.17(0.78,1.69)	71.4 (0.046)	77.4	6.0 (−3.0,15.0)
Tumor size (mm)							
0–20	547	51	39	1.31(0.97,1.72)	90.7 (0.012)	94.2	**3.5 (1.1,5.9)**
21–50	813	170	102	**1.67(1.43,1.94)**	79.1 (0.014)	88.5	**9.4 (6.7,12.1)**
51+	178	65	32	**2.03(1.57,2.59)**	63.5 (0.036)	82.8	**19.3 (12.2,26.4)**
Number of positive nodes							
0	806	72	70	1.03(0.80,1.30)	91.1 (0.010)	92.4	1.3(−0.7,3.3)
1–3	389	83	46	**1.80(1.44,2.24)**	78.7 (0.021)	89.4	**10.7(6.6,14.8)**
4–9	192	64	30	**2.13(1.64,2.72)**	66.7 (0.034)	85.8	**19.1(12.4,25.8)**
10+	123	61	23	**2.65(2.03,3.41)**	50.4 (0.045)	82.3	**31.9(23.1,40.7)**
Unknown	28	6	4	1.50(0.55,3.26)	78.6 (0.078)	90.6	12.0 (−3.3,27.3)
ER status							
Negative	528	146	73	**2.00(1.69,2.35)**	72.3 (0.019)	87.2	**14.9(11.2,18.6)**
Positive	850	99	82	1.21(0.98,1.47)	88.4 (0.011)	91.7	**3.3 (1.1,5.5)**
Unknown	160	41	18	**2.28(1.63,3.09)**	74.4 (0.035)	89.8	**15.4 (8.5,22.3)**
PR status							
Negative	423	106	57	**1.86(1.52,2.25)**	74.9 (0.021)	87.4	**12.5(8.4,16.6)**
Positive	586	73	58	1.26(0.99,1.58)	87.5 (0.014)	91.6	**4.1 (1.4,6.8)**
Unknown	529	107	58	**1.84(1.51,2.23)**	79.8 (0.017)	90.2	**10.4 (7.1,13.7)**
Her2 status							
Negative	665	78	68	1.15(0.91,1.43)	88.3 (0.012)	91.1	**2.8 (0.4,5.2)**
Equivocal	35	7	4	1.75(0.70,3.61)	80.0 (0.068)	89.9	9.9 (−3.4,23.2)
Positive	418	84	53	**1.58(1.26,1.96)**	79.9 (0.020)	87.9	**8.0 (4.1,11.9)**
Unknown	420	117	48	**2.44(2.02,2.92)**	72.1 (0.022)	89.7	**17.6 (13.3,21.9)**
Histology							
Ductal	1346	270	155	**1.74(1.54,1.96)**	79.9 (0.011)	89.6	**9.7 (7.5,11.9)**
Lobular	71	7	7	1.00(0.40,2.06)	90.1 (0.035)	91.0	0.9 (−6.0,7.8)
Mucinous	58	1	4	0.25(0.01,1.39)	98.3 (0.017)	96.0	−2.3 (−5.6,1.0)
Others	63	8	7	1.14(0.49,2.25)	88.9 (0.040)	89.7	0.8 (−7.0,8.6)
Grade							
1	161	8	11	0.73(0.31,1.43)	95.0 (0.017)	95.6	0.6 (−2.7,3.9)
2	661	111	71	**1.56(1.29,1.88)**	83.2 (0.015)	90.5	**7.3 (4.4,10.2)**
3	433	119	59	**2.02(1.67,2.41)**	72.5 (0.021)	87.7	**15.2 (11.1,19.3)**
Unknown	283	48	32	**1.50(1.11,1.99)**	83.0 (0.022)	89.8	**6.8 (2.5,11.1)**
Chemo-therapy							
No chemo-therapy	440	58	53	1.09(0.83,1.41)	86.8 (0.016)	90.4	**3.6 (0.5,6.7)**
1^st^ Gen	162	49	21	**2.33(1.73,3.08)**	69.8 (0.036)	88.1	**18.3 (11.2,25.4)**
2^nd^ Gen	915	174	97	**1.79(1.54,2.08)**	81.0 (0.013)	90.0	**9.0 (6.5,11.5)**
3^rd^ Gen	21	5	2	2.50(0.81,5.83)	76.2 (0.093)	90.8	14.6 (−3.6,32.8)
Hormone-therapy							
No	398	108	51	**2.12(1.74,2.56)**	72.9 (0.022)	87.7	**14.8 (10.5,19.1)**
Yes	1140	178	122	**1.46(1.25,1.69)**	84.4 (0.011)	90.8	**6.4 (4.2,8.6)**

Numbers marked in bold indicate statistically significant difference at the 95% confidence level

### Nodal status calculator

**Fig. 1:**
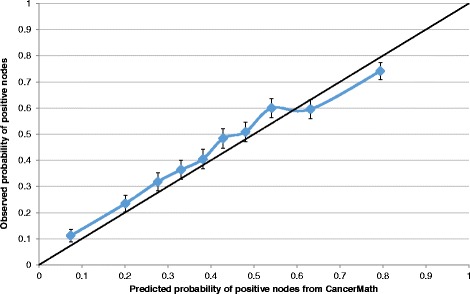
Calibration plot of observed probability of positive nodes with 95 % confidence interval against predicted probability of positive nodes (mean) by deciles of the predicted value

A total of 6807 patients with nodal status data were selected for validation of nodal status calculator. In this dataset, 43.6 % patients (*n* = 2970) (95 % CI, 42.5 %–44.8 %) had at least one positive lymph node and the median predicted probability was 40.6 %. CancerMath underestimated the probability of positive node for most of the subgroups (Table 1). The calibration plot (Fig. 1) also illustrated underestimation except for the last two deciles. The discrimination of this calculator was fair, with AUC of 0.71 (95 % CI, 0.70–0.72).

### Outcome calculator

**Fig. 2:**
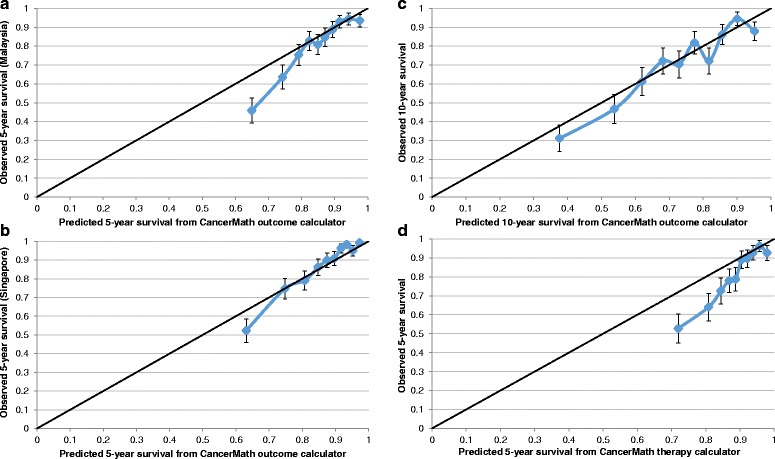
Calibration plot of observed survival with 95 % confidence interval against predicted survival (mean) by deciles of the predicted value. **a** 5-year survival from outcome calculator for Malaysian patients, **b** 5-year survival from outcome calculator for Singaporean patients, **c** 10-year survival from outcome calculator, **d** 5-year survival from therapy calculator

**Fig. 3:**
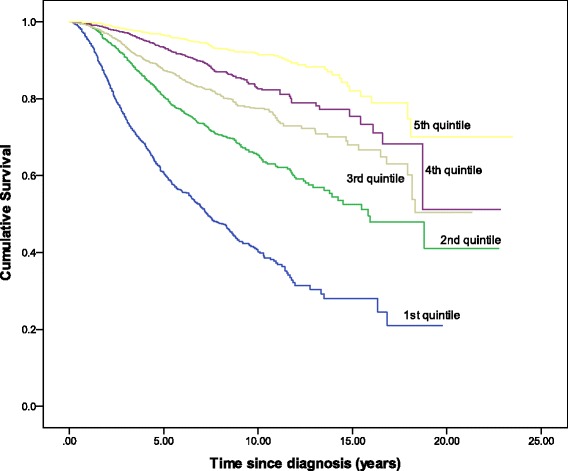
Kaplan-Meier curves of overall survival by quintiles of 5-year predicted survival from outcome calculator

The observed number of deaths within 5 years after diagnosis was significantly higher than the predicted number of deaths (752 vs 667, MR = 1.13, 95 % CI 1.05–1.21). The number of observed and predicted number of deaths within 10 years after diagnosis was not significant (488 vs 454, MR = 1.07, 95 % CI 0.98–1.17). The absolute differences of 5-year and 10-year predicted and observed survival probabilities were 3.9 % and 4.9 %. Overestimation was more pronounced in Malaysian patients than in Singaporean patients (5.8 % vs 2.5 % for 5-year survival, and 8.0 % vs 0.0 % for 10-year survival). We also observed notable differences for cases diagnosed in earlier period and of younger age (Tables 2 and 3). In addition, CancerMath significantly overpredicted survival for patients with unfavorable prognostic characteristics such as large tumor size, more positive nodes and ER negative tumor. For those with relatively better predicted survival, CancerMath predictions were similar to observed outcome (Fig. 2a, b and c). The difference between 5-year predicted and observed survival was 15 %, 3 % and 1 % for the first, fifth, and tenth deciles respectively. The Kaplan-Meier curves of overall survival by quintiles of predicted 5-year survival were illustrated in Fig. 3. The difference in survival experience between the five groups was statistically significant (*p*-value < 0.001 by the log-rank test). The AUC for 5-year and 10-year overall survival were 0.77 (95 % CI,0.75–0.79) and 0.74 (95 % CI,0.71–0.76), respectively whereas the c-index was 0.74 (95 % CI, 0.72–0.75). Both measures demonstrated fair discrimination.

### Therapy calculator

For therapy calculator which was only validated in Malaysian patients, predicted survival was significantly higher than the observed survival for almost all subgroups, except for those diagnosed recently and with more favorable tumor characteristics (Table 4, Fig. 2d). The calculator showed fair discrimination at 5-year overall survival (AUC = 0.73, 95 % CI 0.70–0.77).

### Conditional survival calculator

For patients who have survived 2 years since diagnosis, the predicted 5-year survival was 91.0 % versus the observed survival of 88.3 %. The AUC was 0.75 (95 % CI, 0.73–0.77). For patients who have survived 5 years and 7 years, the predicted probability of surviving up to 10 years was 86.6 % and 91.7 %. Whereas the observed survival was 85.3 % and 91.0 % correspondingly. The AUC was 0.66 (95 % CI, 0.62–0.70) and 0.63 (95 % CI, 0.57–0.68) for 10-year survival.

## Discussion

Many prognostic tools have been developed over the past two decades to aid clinical decision making for breast cancer patients. This study validated four different prognostic calculators provided by CancerMath in the Singapore-Malaysia Hospital-Based Breast Cancer Registry. The discrimination was fair for nodal status calculator. CancerMath outcome, therapy and conditional survival calculator also moderately discriminated between survivors and non-survivors at 5 years and 10 years after diagnosis. It however consistently overestimated survival for this cohort of Southeast Asian patients, especially for those with poor prognostic profile.

CancerMath was previously built and validated using SEER data and patients diagnosed at Massachusetts General and Brigham and Women’s Hospitals [19]. In the SEER database, 82.7 % of the invasive breast cancer cases diagnosed between 2003 and 2007 were white and only 6.9 % were Asian/ /Pacific Islander [28]. It was shown that the differences between observed and predicted survival was within 2 % for 97 % of the patients in the validation set [19]. Our study is the first one to independently validate CancerMath outside United States and is also the largest validation study of a western-derived breast cancer prognostic model in Asia. We demonstrated that CancerMath overpredicted survival by more than 3 % for almost all clinical and pathological subgroups. The findings were similar to previous validation studies of Adjuvant! Online conducted in Asia. In the Malaysian, Korean, and Taiwanese studies, the predicted and observed 10-year overall survival differed by 6.7 %, 11.1 %, and 3.9 % correspondingly [16–18]. The AUC of Adjuvant! Online was 0.73 (95 % CI, 0.69–0.77) in the Malaysian study and hence very close to the AUC of CancerMath reported in the present study [16]. Furthermore the prediction was too optimistic for young patients in almost all validation studies of Adjuvant! Online [12, 15–17]. Although adjustment of 1.5-fold increase in risk was added to Adjuvant! Online version 7.0 for patients younger than 36 years and with ER positive breast cancer, overprediction was still found in recent validation studies [12, 16, 17]. Our findings from current validation of CancerMath also suggested that correction for young age at diagnosis is needed.

The selection of patients for validation can partially explain the discrepancy in observed and predicted survival. CancerMath has only been validated among patients with tumor size no more than 50 mm and positive nodes no more than seven [29]. In our validation dataset, 10 % of patients had tumor size larger than 50 mm and 8 % had more than ten positive nodes. However even for patients with tumor size in between 20 mm and 50 mm and one to three positive nodes, the difference between the predicted and observed survival was more than 3 %. In general, Asian patients are more likely to present with unfavorable prognostic features such as young age, negative hormone receptor status, HER2 overexpression, and more advanced stage compared to their western counterparts [30–32]. In our current analysis, reduced agreement was observed for patients with poorer predicted outcome, especially for Malaysian patients, as illustrated by the calibration plot. In addition, the slope of the calibration plot for Malaysian patients were greater than 1 for the first three deciles which suggested that the spread of the predicted survival was less than observed survival. CancerMath’s poorer performance in Malaysia might be explained by higher proportion of patients in advanced stages and more heterogeneous prognosis in Malaysia. Such limitation of CancerMath may restrict its use to patients with better prognostic profile only. Furthermore CancerMath therapy calculator applies the same amount of risk reduction from adjuvant therapy as Adjuvant! Online, which was estimated from meta-analysis of clinical trials mainly conducted in western population [9, 19]. However non-adherence to treatment is more common among Asian women [33–35]. Studies also reported different drug metabolism and toxicity induced by chemotherapy between Asian and Caucasian patients [36]. These evidences may imply CancerMath overestimate the effect of treatment in Asian patients.

Another possible explanation of suboptimal performance of CancerMath and also the limitation of our study is missing data on ER (6 %), PR (15 %), HER2 status (47 %), and tumor grade (11 %). For patients with complete information on required predictors (*N* = 1872), the predicted and observed 5-year survival was 86.0 % and 82.5 %. The difference were similar to what we observed in the entire dataset. Therefore the impact of missing data is relatively small on performance of CancerMath.

Several gene expression profiling assays, such as MammaPrint [37] and Oncotype Dx [38] are currently available for breast cancer prognostication and treatment decision. However these tools do not incorporate clinicopathologic factors which are readily available or relatively cheap to obtain. Due to the high cost of these tests and larger proportion of patients with high predicted risk in Asia [39, 40], the clinical utility is uncertain in this region. Therefore traditional prognostic model using clinicopathologic factors seems more reasonable in our local setting.

## Conclusions

In conclusion, CancerMath demonstrated modest discrimination and calibration among Southeast Asian patients. Our results suggest that CancerMath is more suitable for patients diagnosed with favorable disease.

## Abbreviations

AC: Doxorubicin and cyclophosphamide
AI: Aromatase inhibitors
AUC: Area under the curve
CI: Confidence interval
C-index: Concordance index
CMF: Cyclophosphamide, methotrexate and fluorouracil
ER: Estrogen receptor
FEC: Fluorouracil, epirubicin and cyclophosphamide
FISH: Fluorescence in situ hybridization
HER2: Human epidermal growth factor receptor 2
IHC: Immunohistochemistry
MR: Mortality ratio
NUH: National University Hospital
PR: Progesterone receptor
SEER: Surveillance, epidemiology and end-result
TAC: Docetaxel, doxorubicin and cyclophosphamide
TTSH: Tan Tock Seng Hospital
UMMC: University Malaya Medical Centre.
